# Bioactive Molecules from the Invasive Blue Crab *Callinectes sapidus* Exoskeleton: Evaluation of Reducing, Radical Scavenging, and Antitumor Activities

**DOI:** 10.3390/md23010045

**Published:** 2025-01-17

**Authors:** Francesco Longo, Alessandro Attanzio, Laura Marretta, Claudio Luparello, Serena Indelicato, David Bongiorno, Giampaolo Barone, Luisa Tesoriere, Ilenia Concetta Giardina, Giulia Abruscato, Manuela Perlotti, Lucie Branwen Hornsby, Vincenzo Arizza, Mirella Vazzana, Aiti Vizzini, Chiara Martino, Angelica Listro, Vinicius Queiroz, Antonio Fabbrizio, Paolo Salvatore Francesco Ciaccio, Stella Maria Cascioferro, Francesca Di Gaudio, Manuela Mauro

**Affiliations:** 1 Department of Biological, Chemical and Pharmaceutical Sciences and Technologies (STEBICEF), University of Palermo, 90123 Palermo, Italy; francesco.longo03@unipa.it (F.L.); alessandro.attanzio@unipa.it (A.A.); laura.marretta02@unipa.it (L.M.); claudio.luparello@unipa.it (C.L.); david.bongiorno@unipa.it (D.B.); giampaolo.barone@unipa.it (G.B.); luisa.tesoriere@unipa.it (L.T.); ileniaconcetta.giardina@unipa.it (I.C.G.); giulia.abruscato@unipa.it (G.A.); manuela.perlotti@community.unipa.it (M.P.); lbhornsby@gmail.com (L.B.H.); mirella.vazzana@unipa.it (M.V.); aiti.vizzini@unipa.it (A.V.); chiara.martino@unipa.it (C.M.); listroangelica@gmail.com (A.L.); stellamaria.cascioferro@unipa.it (S.M.C.); manuela.mauro01@unipa.it (M.M.); 2 National Biodiversity Future Center (NBFC), Piazza Marina 61, 90133 Palermo, Italy; 3 Departamento de Fisiologia, Instituto de Biociências, Universidade de São Paulo, Sao Paulo 05508-090, Brazil; vinicius_ufba@yahoo.com.br; 4 Department of Theoretical and Applied Sciences (DiSTA), University e Campus, 22060 Novedrate, Italy; antonio.fabbrizio@uniecampus.it; 5 Independent Researcher, 92019 Sciacca, Italy; paciaccio@libero.it; 6 Department PROMISE, University of Palermo, Piazza delle Cliniche, 2, 90127 Palermo, Italy; francesca.digaudio@unipa.it

**Keywords:** Atlantic blue crab, crustaceans, exoskeleton, chitosan, astaxanthin, polyphenols, antitumor, antioxidant, reducing capacity, radical scavenger activity

## Abstract

In recent years, the invasive Atlantic blue crab (*Callinectes sapidus*) has increased its spread throughout the Mediterranean Sea, threatening native biodiversity and local economies. This study aimed to valorize *C. sapidus* sampled in Sicily by utilizing its exoskeleton as a source of chitosan, astaxanthin, and bio-phenolic compounds. These biomolecules were evaluated for their reducing, radical scavenging, and antitumor activity. The ferric ion reducing antioxidant power (FRAP) and the free radical scavenging activity against radical 2,2-Diphenyl-1-picrylhydrazyl (DPPH) were significantly higher for chitosan (3.16 ± 0.10 mg AAE/g and 8.1 ± 0.10 µmol TE/g). No significant differences were observed among the tested biomolecules in their activity in scavenging the radical 2,2′-azino-bis (3-ethylbenzothiazoline-6-sulfonic acid) (ABTS). Both bio-phenolic compounds and astaxanthin exhibited dose-dependent cytotoxicity on CaCo-2 (IC_50_ = 12.47 and 18 µg/mL) and HepG2 (IC_50_ = 10.25 and 1.26 µg/mL) cell lines, while only bio-phenols showed no cytotoxic effect on differentiated CaCo-2 cells up to 20 µg/mL. These findings highlight the value of blue crab by-products in supporting a circular economy, offering a sustainable approach to managing this invasive species while providing bioactive compounds with promising medical and nutraceutical applications.

## 1. Introduction

The Atlantic blue crab, *Callinectes sapidus* (Rathbun, 1896), is a decapod crustacean belonging to the Portunidae family, characterized as a eurythermal and euryhaline species able to inhabit estuaries, lagoons, and coastal waters at depths of up to 90 m [1,2]. *C. sapidus* is an opportunistic predator that feeds on a wide range of organisms and exhibits a high level of fecundity, aggressive behavior, and excellent swimming abilities [3,4]. This species, originally from the Western Atlantic Ocean, has rapidly expanded its distribution in recent years [5]. In the Mediterranean Sea, it is considered an alien and invasive species, classified among the 666 non-native species reported in the latest monitoring report by Zenetos and Galanidi [6]. The first recorded sighting of *C. sapidus* in the Mediterranean Sea dates back to 1949, with documented reports in the waters of the Northern Adriatic [7,8], probably introduced via the ballast water of ships [2]. *C. sapidus* populations have since grown steadily with a progressive expansion across the Mediterranean basin [9].

Key factors contributing to this spread include climate change, which has made the Mediterranean waters increasingly hospitable to tropical and subtropical species [10] and the intensification of maritime traffic, which likely facilitated additional introductions of the species from its native range [11]. Today, thanks to its physiological traits, such as its high level of tolerance to salinity and temperature fluctuations, the blue crab has successfully established itself across almost the entire Mediterranean Sea [5,9]. Since 2023 an unprecedented spread of *C. sapidus* in the Northern Adriatic has posed a serious threat to local ecosystems and severely compromised shellfish farms, particularly widespread in this region, causing damage currently estimated to cost around 100 million EUR [12]. The species has also been reported in abundance on the Sicilian coast, with initial reports dating back to the 1970s [13] and with a notable increase in recent years [14]. On this island, the species is distributed unevenly along the whole coastline [14] with an increase in sightings around brackish coastal lagoons and river mouths [15,16,17,18,19].

Considering that *C. sapidus* is highly valued for human consumption in its native range [9] and is known for the high nutritional quality of its meat [20,21,22], one of the main strategies proposed to contain and monitor its spread in Italy is its utilization for food purposes [9], as already implemented in other Mediterranean regions, such as Greece, Turkey, and Tunisia [5,20,23]. To this end, in January 2024, *C. sapidus* was officially included in the Italian list of fish species of commercial interest via a ministerial decree [24]. It is important to note that the waste portion of the blue crab, primarily consisting of the exoskeleton, accounts for up to 80–85% of the total weight [25,26]. This biomass when treated as a by-product can be highly valorized through the extraction of bioactive molecules with pharmaceutical and nutraceutical properties [27,28,29,30,31]. This approach is supported by numerous studies investigating the bioactive potential of by-products from fish processing and highlighting how these can become valuable sources for nutraceutical and pharmaceutical applications. Examples include gelatin and antioxidant peptides from skipjack tuna (*Katsuwonus pelamis*) skins [32], gelatin from Siberian sturgeon (*Acipenser baerii*) cartilage [33], angiotensin-I-converting enzyme (ACE) inhibitory peptides from the collagen of monkfish (*Lophius litulon*) swim bladders [34], and antioxidant peptides obtained from the protein hydrolysates of skate cartilage (*Raja porosa*) [35] or of bluefin leatherjacket (*Navodon septentrionalis*) skin [36].

The exoskeleton of crustaceans is known to be composed mainly of proteins (20–40%), chitin (15–40%), minerals (20–50%), and small quantities of lipids and pigments, in varying amounts depending on the species and season [31].

Chitin is one of the most abundant polysaccharides in nature, composed of N-acetyl-D-glucosamine units linked together by β-1,4 glycosidic bonds [37]. Although non-toxic, biocompatible, and biodegradable due to its insolubility in most common solvents, this polymer is difficult to use [38,39]. This limitation can be overcome through the deacetylation of its N-acetyl groups to obtain chitosan [40]. This polysaccharide has applications in various fields, including wastewater treatment [41], agriculture [42], the food industry [43], and especially in the pharmaceutical and biomedical field, in which it is widely valued for its antimicrobial, antifungal, anti-oxidant, anti-inflammatory, and anticancer activities [44,45]. Another biomolecule of considerable interest that can be extracted from the exoskeletons of crustaceans is astaxanthin [46]. This carotenoid is one of the most powerful natural antioxidants [47,48,49]. This molecule shows anti-cancer efficacy against various types of cancer, such as those of the mouth, bladder, colon, and hepatocellular carcinoma [50]. It is widely used in nutraceutical and cosmetic products, available in the form of capsules, soft gels, tablets, powder, biomass, cream, energy drinks, oil, and extract [46]. Additionally, both the Food and Drug Administration and the European Commission have approved the use of astaxanthin as a food additive in animal feed and fish food [51,52,53]. Crustacean exoskeletons also contain another class of molecules known for their notable antioxidant properties: phenolic compounds. These compounds are categorized based on their structure into phenolic acids, stilbenes, and flavonoids (including flavanols, flavones, flavanones, isoflavones, flavan-3-ols, and anthocyanins) [54]. Besides being potent antioxidants, these compounds exhibit a wide range of biological activities, such as antitumor, anti-inflammatory, antihypertensive, antidiabetic, antimicrobial, antiviral, algicidal, antifungal, and insecticidal effects [55,56]. While phenolic compounds have been studied predominantly in plants, recent research has been exploring the phenolic composition of aquatic organisms, particularly crustaceans [57,58].

This study aimed to extract chitosan, astaxanthin, and bio-phenolic compounds from the exoskeletons of *C. sapidus* captured in Sicily, evaluating their yield, quality, reducing capacity, and scavenging activity. The cytotoxic effects of the astaxanthin and polyphenol extracts were also assessed on human cancer and differentiated cell lines cultured in vitro. These findings could contribute to strategies for managing the spread of *C. sapidus*. In addition to the use of its meat for food, valorizing its exoskeletons as a source of bioactive molecules with pharmaceutical potential may help contain the spread of this species while mitigating its environmental impact.

## 2. Results

### 2.1. Exoskeleton Yield and Humidity

The specimens in this study had an average total weight of 173.5 ± 36.9 g and an average exoskeleton weight of 60.7 ± 8.4 g. The dry exoskeleton powder was obtained with a yield of 14.4 ± 0.4%. The exoskeletons showed a percent humidity of 61.0 ± 1.7%.

### 2.2. Chitosan Characterization

**Figure 1:**
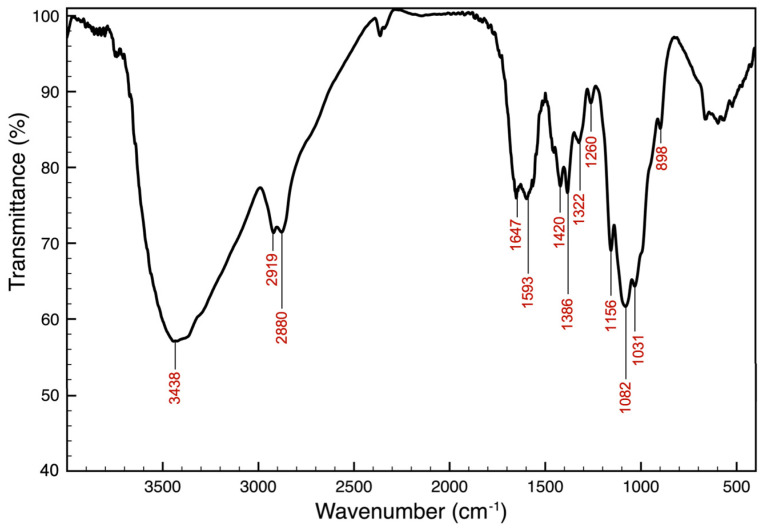
FT-IR spectrum of chitosan obtained from *C. sapidus* exoskeleton.

During chitosan preparation, the chitin average yield was 15.66 ± 1.25%, and that of the chitosan was 10.71 ± 0.48% before purification and 7.93 ± 0.34% after purification. The chitosan spectrum, obtained using a Fourier transform infrared (FT-IR) spectroscopy analysis, is shown in Figure 1 and revealed characteristic peaks corresponding to chitosan functional groups.

The peaks at 3438 cm^−1^ were related to the stretching of O-H and N-H bonds, whereas the peaks at 2919 and 2880 cm^−1^ were attributed to the stretching of C-H bonds. The absorption at 1647 cm^−1^ corresponds to the amide I band (the stretching of the C=O group), while the peak at 1593 cm^−1^ is related to the amide II band (the bending of the N-H bonds of NH_2_ and CONHR groups). The bands at 1420, 1386, and 1322 cm^−1^ are attributed to the bending of the C-H bonds of the CH_3_, CH_2_, and CH groups, respectively. The absorption at 1260 cm^−1^ is associated with the stretching vibrations of the C-N bonds, whereas the peaks at 1156 and 1082 cm^−1^ are attributable to the stretching of the C-O-C bonds (the glycosidic bond and bond between anomeric C_1_ and O in the pyranose ring). The peak at 1031 cm^−1^ is characteristic of the stretching vibrations of the C-OH bonds, and the band at 898 cm^−1^ is associated with the wagging of the chitosan saccharide skeleton. The calculated degree of acetylation (DA%) of the extracted chitosan was 10.8%, resulting in a DDA% of 89.2%.

### 2.3. Astaxanthin Identification and Quantification

The chromatogram of ethanolic extracts of *C. sapidus* exoskeleton powder displayed a peak with a retention time of 13.58 min, which can reasonably be attributed to astaxanthin.

**Figure 2:**
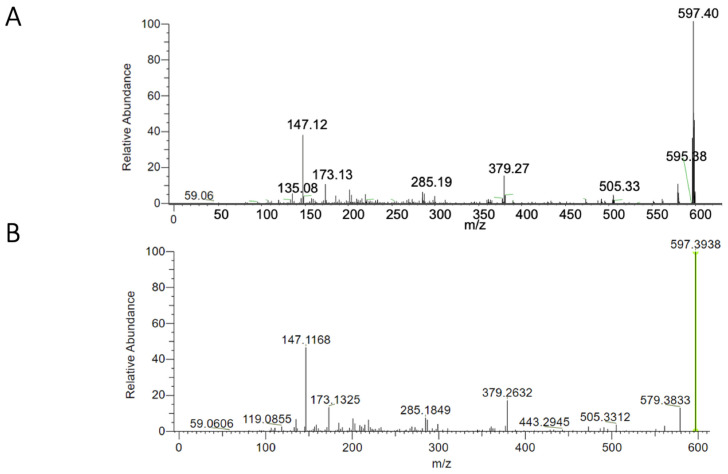
Spectrum of astaxanthin of *C. sapidus* (**A**) and spectrum reported on the MZCloud database (**B**). The green line represents *m*/*z* ratio of [M+H]^+^ astaxanthin precursor ion on database.

The tandem mass spectrometry (MS/MS) spectrum of precursor ions at 597.3938 (*m*/*z*), with the formula C_40_H_52_O_4_, acquired under the same experimental conditions as those reported for astaxanthin in the publicly available MZCloud database, showed a fragmentation pattern that was superimposable on the reference spectrum (Figure 2). An analysis by UPLC-HRMS confirmed the presence of astaxanthin in the ethanolic extract with a yield of 52.0 ± 6.0 μg/g of the exoskeleton powder of *C. sapidus*.

### 2.4. Polyphenol Compounds’ Identification and Quantification

The total phenolic content (TPC) evaluated in the phenolic extract of the exoskeleton of *C. sapidus* was 3.4 ± 0.9 mg GAE/g.

The bioactive phenolic compounds identified using the ultra-high performance liquid chromatography tandem mass spectrometry (UPLC–MS) analysis are summarized in Table 1. Mandelic acid was detected in the highest concentration, while coumaric acid, ferulic acid, rutin, trans-hydroxycinnamic acid, and luteolin were present in lower amounts. Gentisic acid was also identified, albeit in concentrations below the limit of quantification.

### 2.5. Reducing Capacity and Radical Scavenging Activity

**Figure 3:**
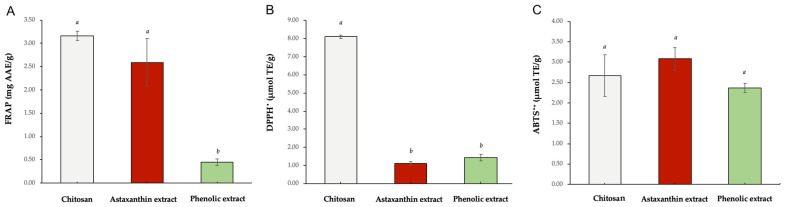
Reducing (**A**) and radical scavenging activity (**B**,**C**) of chitosan, astaxanthin, and phenolic extract of *C. sapidus* exoskeleton. AAE: acid ascorbic equivalent; TE; trolox equivalent. Values are expressed as a mean ± SD. Bars within the same graph with different letters are significantly different for *p* < 0.05.

The reducing capacity and radical scavenging activity are summarized in Figure 3. In the FRAP assay (Figure 3A), a significantly higher reducing capacity value was observed for chitosan (3.16 ± 0.10 mg AAE/g) compared to both the astaxanthin extract (2.59 ± 0.51 mg AAE/g) and the phenolic extract (0.45 ± 0.07 mg AAE/g). In the DPPH test (Figure 3B), chitosan showed an activity of 8.1 ± 0.10 µmol TE/g, which was significantly higher than that of the astaxanthin extract and the phenol extract, which exhibited 1.12 ± 0.08 and 1.43 ± 0.18 µmol TE/g, respectively. No significant changes were observed in any of the samples following the ABTS assay (Figure 3C); however, a slightly higher level of activity was found in the astaxanthin extract (3.08 ± 0.28 µmol TE/g) than the chitosan (2.67 ± 0.51 µmol TE/g) and phenolic extracts (2.37 ± 0.11 µmol TE/g).

### 2.6. In Vitro Cytotoxic Activity on Human Cancer and Differentiated Cells

**Figure 4:**
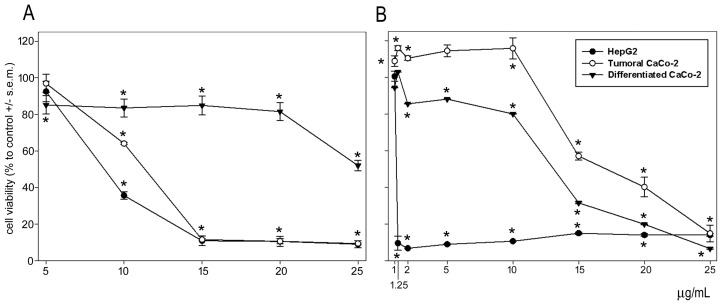
Dose-response effect of polyphenolic extract (**A**) and astaxanthin (**B**) from *C. sapidus* exoskeleton on the viability of HepG2 and CaCo-2 cells, both tumoral and differentiated, after 24 h of exposure. The error bars correspond to the standard error of the mean (s.e.m.) of three independent measurements. * *p* < 0.05.

The cytotoxic effects of both the astaxanthin and polyphenolic extracts on HepG2 hepatocarcinoma and CaCo-2 colorectal cancer and differentiated cells were examined using an MTT assay. Figure 4A shows that the polyphenolic extract induced a comparable dose-dependent decrease in viability in both cell lines, with mean IC_50_ values of 10.25 µg/mL (HepG2) and 12.47 µg/mL (CaCo-2). Interestingly, the polyphenols failed to exert a prominent cytotoxic effect on the differentiated CaCo-2 cells up to a concentration of 20 µg/mL, thereby suggesting their potential for the development of novel specific treatment agents against colon cancer. On the other hand, HepG2 cells were much more sensitive than CaCo-2 cells to the action of astaxanthin after exposure for 24 h, with mean IC_50_ values of 1.26 µg/mL (HepG2) vs. 18 µg/mL (CaCo-2). Astaxanthin also exerted a dose-dependent cytotoxic effect on differentiated CaCo-2 cells with a mean IC_50_ of 13.3 µg/mL (Figure 4B).

## 3. Discussion

The Atlantic blue crab (*C. sapidus*), present in the Mediterranean for over 70 years [2,8], has undergone an unprecedented numerical expansion in Italy since 2023 [12]. To manage its invasion in affected areas and prevent its spread to regions not yet severely afflicted, innovative strategies are required. In addition to its use for human consumption, the exoskeleton of *C. sapidus* could be a valuable resource for the extraction of bioactive molecules [27,28,59,60,61,62]. In this study, exoskeletons of *C. sapidus* collected in Sicily were used to obtain bioactive molecules, such as chitosan, astaxanthin, and bio-phenolic compounds. Their yield, quality, reducing capacity, radical scavenging activity, and in vitro antitumor potential were evaluated. The exoskeleton, a significant non-edible portion, accounted for 37% of the total animal weight. The exoskeleton was processed into a dry powder with a yield of 14 ± 0.4%. Using a chemical extraction method on this powder, both chitin and chitosan were successfully extracted, chitin was obtained with a yield of 15.66 ± 1.25%, and chitosan was produced through subsequent alkaline deacetylation with a yield of 10.71 ± 0.48%. The lower chitosan yield compared to chitin is partially explained by the replacement of N-acetyl groups with amino groups during the deacetylation process [63]. To obtain extremely pure chitosan suitable for potential biomedical applications [64], an additional purification step was implemented, resulting in a final purified chitosan yield of 7.93 ± 0.34%. The chitin and chitosan yields from *C. sapidus* collected in Sicily were slightly higher than those reported by Metin et al. [65] and Kaya et al. [66] for *C. sapidus* regarding the Köyceğiz lagoon (Muğla, Turkey) and İskenderun (Antakya, Turkey), respectively. These studies reported chitin yields of 10.83% and 12.1% and unpurified chitosan yields of 7.47% and 9.2%.

The chitosan we extracted was characterized using Fourier transform infrared (FTIR) spectroscopy, determining a degree of deacetylation (DDA) of 89.2%. This value exceeds those reported by Metin et al. [65] and Kaya et al. [66], who recorded DDAs of 77% and 82.5%, respectively. The DDA is significantly influenced by the chitin extraction method and conditions used during the deacetylation process, including alkali concentration, temperature, and reaction time [38]. This parameter is crucial, as it directly affects the physicochemical properties and biological activities of chitosan [67]. Higher DDA values are particularly desirable, as they enhance the biocompatibility of chitosan [68]. The chitosan obtained in this study, with a DDA of 89.2%, falls well within the recommended range for medical applications, which is between 85% and 95% [64,69].

Another biomolecule of known commercial interest, present in the exoskeleton of *C. sapidus*, is astaxanthin [70,71]. In the current study, using an ultrasound-assisted solvent extraction technique, 52.0 ± 6.0 μg of astaxanthin per gram of dry exoskeleton powder was obtained from *C. sapidus* captured in Sicily. This astaxanthin yield was higher than the yields of other studies conducted on *C. sapidus* using traditional solvent extraction methods [59,72,73] and slightly lower than the yields obtained using enzymatic [59] or enzymatic/chemical [72] pretreatments. The amount of extractable astaxanthin is related not only to the amount of carotenoids in the raw material, an extremely variable parameter in crustaceans [74,75], but also to the extraction technique. Indeed, it has been demonstrated that demineralization processes [72], enzymatic proteolysis [59,72], and saponification [73] can significantly influence the amount of astaxanthin extracted. Ultrasound-assisted extraction techniques have been reported as effective methods to optimize the extraction of bioactive molecules from both plant and animal matrices [76,77]. Ultrasonic-wave-induced cavitation generates matrix fragmentation, increasing the contact area between solvent and solid particles, thus improving extraction efficiency [78,79]. This technique reduces energy consumption, extraction times, and the amount of solvent required [79,80].

In addition to proteins, chitin, minerals, lipids, and pigments, a class of secondary metabolites with significant antioxidant activity are found in the exoskeleton of crustaceans: phenolic compounds [57,58,81,82]. This class of biomolecules has attracted the interest of the food, nutraceutical, pharmaceutical, and cosmetics industries thanks to their properties [58]. In this study, for the first time, the bio-phenolic compounds present in the exoskeleton of *C. sapidus* were qualitatively and quantitatively characterized, and their reducing capacity, scavenging, and antitumor activities in vitro were evaluated. Seven bio-phenolic compounds were identified, six of which were within the limit of quantification (LOQ), including mandelic acid, found to be present in the highest concentration. This compound is an aromatic alpha hydroxy acid that possesses interesting antioxidant and antimicrobial properties. It is also widely used in cosmetic products due to its exfoliating and antibacterial properties, which are useful for treating acne [83]. The total phenol content (TPC) of the extract in our study was measured as 3.4 ± 0.9 mg GAE/g using the Folin–Ciocalteu reaction. This was higher than the findings of Onodenalore et al. [57] on ethanolic extracts of *Pandalus borealis*, slightly lower than those of Maia et al. [81] on ethanolic extracts of exoskeletons of *Palaemon serratus* and *Palaemon varians*, and lower than the TPC obtained by Pereira et al. [58] on aqueous and ethanolic extracts of the exoskeletons of *Litopenaeus vannamei* and of *Ucides cordatus*. The observed differences in TPC values may be attributed to several variables known to influence the phenolic content in the exoskeletons of crustaceans. These include factors such as species, geographic location, collection period, and extraction method, all of which can significantly affect the TPC value in phenolic extracts from crustaceans [58,81].

Our results demonstrated that all the tested molecules exhibited significant activity, both in terms of reducing capacity and radical scavenging activity. In particular, chitosan showed significantly higher FRAP and DPPH values than the other biomolecules and also noteworthy ABTS^•+^ radical reduction activity. Chitosan’s antioxidant activity primarily arises from its hydroxyl (-OH) and amino (-NH_2_) groups [84]. The high levels of reducing and radical scavenging activity, as observed in the present study, are consistent with chitosan’s high DDA value; in fact, chitosan antioxidant activity has been shown to increase with a higher degree of deacetylation [85]. Previous studies have investigated the radical scavenging activity of chitosan obtained from the exoskeleton of *C. sapidus* using DPPH and ABTS assays, expressing the results in terms of radical formation inhibition rates [65,66]. Although our results are not explicitly framed in these terms, comparisons with Trolox, employed as a reference standard, reveal a strong alignment with findings reported in the literature. This consistency not only validates our data but also highlights their relevance and robustness within the context of established antioxidant evaluation methodologies, further supporting the scientific credibility of our approach.

The astaxanthin extract showed significantly higher levels of radical scavenging activity in ABTS assay than the other biomolecules. Furthermore, our results demonstrated good reducing and radical scavenging activities in FRAP and DPPH assays. These findings are consistent with those of Abd El-Ghany et al. [59], who previously reported excellent antioxidant activity in scavenging DPPH or ABTS free radicals by astaxanthin extracted from *C. sapidus*. These findings are also consistent with previous reports regarding astaxanthin antioxidant activity [48,49,86,87,88]. Its antioxidant capacity is attributed to a central polyene chain capable of donating electrons, together with keto (C=O) and hydroxyl (-OH) end groups that can donate hydrogen atoms [89].

The phenolic extract also demonstrated good scavenging activity in the DPPH and ABTS assays, showing comparable activity levels to astaxanthin in the DPPH assay and to both molecules in the ABTS assay, though it exhibited a lower capacity to reduce ferric ions compared to other biomolecules in the FRAP assay. Our data compared favorably with those of Maia et al. [81], who assessed the reducing capacity of phenolic extracts from *P. serratus* and *P. varians*. Our results showed superior radical scavenging activity in the DPPH assay but slightly lower activity levels in the ABTS and FRAP assays. Phenolic compounds owe their antioxidant activity primarily to hydroxyl groups, which, by donating hydrogen atoms to the free radicals, prevent their propagation phase [90].

Cytotoxic assays on HepG2 and CaCo-2 cells demonstrated that both astaxanthin and the polyphenolic preparation from *C. sapidus* were able to decrease cell viability in a dose-response manner. Moreover, the polyphenols were not cytotoxic on differentiated CaCo-2 cells, representing an added value in possible future applications of the preparation. Consistent with our results, data from Messina et al. [91] and Tan et al. [92] showed the anti-proliferative effect of astaxanthin from *Parapeneus longirostris* and *Haematococcus pluvialis* on HepG2 cells. This effect is ascribable to the apoptosis-promoting effects of c-Jun and c-Fos down-regulation and p53 up-regulation, as well as the activation of reactive oxygen species. The viability of CaCo-2 cells was also proven to be reduced by astaxanthin extracted from *Xanthophyllomyces dendrorhous* [93]. Various components found in the polyphenolic extract may be responsible for this cytotoxic effect. Literature reports have demonstrated the reduction in viability, impairment of the cell cycle, and promotion of apoptosis induced by ferulic acid and p-coumaric acid on HepG2 and CaCo-2 cells [94,95,96,97]. Gentisic-acid-rich extracts, rutin, and luteolin were found to exert a cytotoxic effect on the cell lines under study, in some cases via the stimulation of autophagy [98,99,100,101].

## 4. Materials and Methods

### 4.1. Samples and Exoskeleton Powder Production

In the summer of 2023, 60 adult individuals of *C. sapidus* were collected in the San Teodoro area (near the Stagnone of Marsala), located on the western coast of Sicily. The specimens were transported whilst alive to the STEBICEF Department (University of Palermo), where they were euthanized using thermal shock (−20 °C for 10 min), weighed, and dissected to collect the exoskeleton. The exoskeletons were weighed, washed with distilled water, and subsequently dried in an oven at 60 °C for 18 h. The dried sample was then ground and sieved to obtain a fine powder with homogeneous particle size.

The exoskeleton powder yield was obtained using the following formula:

(1) $$Powder\;yield\;\%=\frac{\;Powder\;weight\;\times 100}{Total\;weight\;of\;animals}$$

The humidity percentage of the exoskeleton was obtained using the following formula:

(2) $$Humidity\;\%=100-\left(\frac{dry\;weight\times 100}{wet\;weight}\right)$$

where *dry weight* refers to the weight of the exoskeletons after reaching constant weight over time following oven drying, and *wet weight* refers to the weight of the exoskeletons before the drying phase.

### 4.2. Chitosan Preparation and Characterization

Chitosan was obtained from the exoskeleton powder using a modified version of the protocol used by Mauro et al. [102]. This protocol includes three key steps for chitin extraction (deproteinization, demineralization, and decolorization), followed by chitin deacetylation to obtain chitosan. To optimize the yield and quality of the final products, the reaction conditions in terms of temperature, time, and reagent concentrations were modified.

To remove the protein fraction, the exoskeleton powder was suspended in a 3% NaOH solution with gentle continuous stirring for 2 h (1:10 *w*/*v*) at 70 °C. The solution was then cooled to room temperature and centrifuged. The pellet was washed three times with distilled water and dried for 18 h in an oven at 60 °C. During demineralization, the dried deproteinized powder was suspended in a 1 M HCl solution (1:15 *w*/*v*) for 1 h at room temperature; the suspension was then centrifuged and the pellet rinsed three times and dried. This phase was then followed by decolorization: the powder was treated with a 2.5% NaClO solution (1:10 *w*/*v*) for 30 min at room temperature and chitin was obtained. To produce chitosan, the chitin was deacetylated using a 50% NaOH solution (1:15 *w*/*v*) and stirred gently and continuously for 48 h at 100 °C. The suspension was then cooled at room temperature and centrifuged, and the resulting solid residue was washed with distilled water until a neutral pH was achieved. After drying at 60 °C for 18 h, the resulting chitosan was purified by dispersing the dry powder in a 0.5 M acetic acid solution (1:100 *w*/*v*) and stirring vigorously for 24 h at room temperature. The dispersion was filtered, and the filtrate was adjusted to pH of 8 using 10% (*w*/*w*) NaOH to obtain purified chitosan. The suspension was centrifuged at 4000 rpm for 10 min at room temperature, and the resulting pellet was collected and washed three times with an ethanol/water solution (70/30). Finally, the purified chitosan was dried, weighed, and stored for subsequent assays.

The final yield of chitin and chitosan was calculated using the following equation:

(3) $$Yield\;\%=\frac{Dry\;product\;weight\times 100}{Exoskeleton\;powder\;weight\;}$$

The IR spectrum of chitosan was recorded using a Jasco FT/IR 420 (Jasco, Easton, MD, USA) infrared spectrometer. The sample was prepared with KBr and the spectrum was measured in the range of 4000–300 cm^−1^. Transmittance (T) values at 1320 and 1420 cm^−1^, converted to absorbance (A) values, were used to calculate the percentage degree of acetylation (*DA*) of chitosan, using the following equation [103]:

(4) $$DA\%=\frac{\left[\left(A1320/A1420\right)-0.3822\right]}{0.03133}$$

Subsequently, the percentage degree of deacetylation (*DDA*) of chitosan was calculated using the following equation:

(5) DDA%=100−DA%

### 4.3. Astaxanthin Extraction

An ultrasonic-assisted solvent extraction method was used to extract astaxanthin from the exoskeleton powder using a modified version of the protocols developed by Hu et al. [104] and Sharayei et al. [105]. The exoskeleton powder was suspended in absolute ethanol at a 1:4 ratio (*w*/*v*). The suspension was stirred gently for 1 h at room temperature and then sonicated on ice using a VC505 ultrasonic generator (Sonics & Materials Inc., Newtown, CT, USA) operating at a frequency of 20 kHz and a power output of 300 W for 30 min. The suspension was then centrifuged at 2000× *g* for 10 min at 4 °C, and the supernatant was collected and filtered. Subsequent extractions were performed until the solvent became colorless, with three repetitions in total. All steps were performed in the dark.

#### Astaxanthin Determination by UPLC-HRMS (Ultra-Performance Liquid Chromatography–High Resolution Mass Spectrometry)

UPLC-HRMS analysis of the ethanolic extracts derived from exoskeleton powder was performed using a UPLC Ultimate 3000 RS (Dionex, Sunnyvale, CA, USA) connected to an HRMS system -Orbitrap Exploris 120, ( ThermoFisher, San Josè, CA, USA). Chromatographic separation was achieved using a Hypersil Gold (ThermoFisher, San Josè, CA, USA) column (50 × 2.1 mm, 1.9 µm) held at 30 °C. The LC method used the following mobile phases: (A) purified water (LC–MS grade, Sigma-Aldrich, Darmstadt, Germany) with 0.1% formic acid (LC–MS grade, Sigma-Aldrich, Darmstadt, Germany) and (B) acetonitrile (LC–MS grade, Sigma-Aldrich, Darmstadt, Germany). The injection volume of the sample was 10 µL.

The flow rate was set to 300 µL/min. The gradient program proceeded as follows: 0–8.0 min, 60% B; 8.0–13.0 min, linear increase to 100% B; 13.0–17.0 min, hold at 100% B; 17.0–17.01 min, linear decrease back to 60% B; and 17.1–20 min, held at 60% B. The mass spectrometer employed a heated electrospray ionization (HESI) source operating in positive ion mode. Mass spectrometry conditions included the following: HESI (+), spray voltage set to 3500 V, auxiliary gas pressure at 50 a.u., capillary temperature of 350 °C, sheath gas pressure at 50 psi, and S-Lens RF level of 70.00 V. Full MS-SIM experiments were conducted under these settings: SIM microscans set at 1, resolution at 17.500, AGC target of 5 × 10^6^, maximum IT of 200 ms, scan range of 500 to 700 *m*/*z*, and profile spectrum data type. For targeted SIM: runtime 0 to 20 min with positive polarity; general inclusion enabled; SIM microscans set to 1; resolution at 60.000; AGC target 5 × 10^6^; maximum IT of 200 ms; MSX count of 1; and an isolation window of 1.0 *m*/*z*. In MS/MS spectra, precursor ion was set at 597.3938 (*m*/*z*), formula was C_40_H_52_O_4_, collision gas was N_2_, resolution was 15.000, AGC target was standard, isolation window was 4.0 *m*/*z*, and collision energy (%) was set at 10, 25, and 35 Volts. An external calibration method was used to obtain a quantification of astaxanthin in ethanolic extracts. Astaxanthin standard solutions (>70%, from Blakeslea Trispora, Sigma Aldrich) were prepared at concentrations of 1000 ppb, 500 ppb, 100 ppb, and 10 ppb in ethanol. The equation of calibration curve was Y = $2.85\times 10^{4}x-5.95\times 10^{5}$, with a linearity correlation coefficient (R^2^) equal to 0.99.

Prior to analysis using the UPLC-HRMS instrument, each sample was prepared using 0.45 µm (13 mm) CLARIFY-PTFE filters (Phenomenex, Torrance, California, USA) and diluted with methanol at a rate of 1:100. The concentration of the recovered astaxanthin was expressed as µg/g exoskeleton powder.

### 4.4. Phenolic Compounds’ Extraction

Phenolic compounds were extracted by suspending 1 g of *C. sapidus* exoskeleton powder in 80% (*v*/*v*) methanol solution at a 1:4 (*w*/*v*) ratio. The process was carried out in the dark at room temperature, stirring continuously for 1 h. The suspension was then sonicated at 40 °C for 30 min in an ultrasonic water bath and centrifuged at 3700× *g* for 10 min. A second extraction cycle was carried out on the solid residue and the supernatants were filtered using 0.45 μm filters (Millipore, Billerica, MA, USA) through a Büchner funnel. The resulting hydromethanolic extract was stored at −20 °C for further analysis.

#### 4.4.1. Total Phenol Content (TPC)

Total phenol content (TPC) was determined using the Folin–Ciocalteu reaction. This involves reducing phosphotungstic–phosphomolybdic acid (Folin–Ciocalteu’s reagent) to form blue reaction products in an alkaline solution [106]. To conduct this analysis, the solvent in the hydromethanolic extract was evaporated with a rotary evaporator, and the resulting residue was freeze-dried to eliminate residual water. The dried samples were reconstituted in appropriate volumes of 5 mM phosphate-buffered saline (PBS) at pH of 7.4 before being used in the experimental analysis following [107]. Aliquots of samples (10–100 μL) were diluted with water to a final volume of 100 μL and mixed with 100 μL of Folin–Ciocalteu reagent (Sigma-Aldrich, Saint Louis, MO, USA) for 5 min, before adding 3 mL of 2% sodium carbonate. The reaction mixture was incubated at room temperature in the dark for 1 h, and absorbance was measured at 700 nm using a Beckman DU640 spectrophotometer (Beckman, Milan, Italy) against a blank without samples. Quantification was based on a standard curve of gallic acid (10–100 μg/mL), and results are expressed as gallic acid equivalents (GAE, mg per g of sample).

#### 4.4.2. Phenolic Compounds’ Determination by UPLC-HESI-MS

Bio-phenols in hydromethanolic extract of *C. sapidus* exoskeleton powder were identified following a modified version of the protocol developed by Indelicato et al. [108], with an optimized UPLC/MS-MS method developed for this study. Analysis was conducted on a Dionex (Sunnyvale, CA, USA) Ultimate 3000 system linked to a ThermoFisher TSQ Quantiva triple-stage quadrupole mass spectrometer (ThermoFisher, San Josè, CA, USA). Chromatographic separation employed a C18 reversed-phase Hypersil GOLD (ThermoFisher, San Josè, CA, USA) analytical column (2.1 × 50 mm, 1.9 μm particle size) maintained at 30 °C, with an injection volume of 5 μL. The separation gradient used mobile-phase (A)—purified water with 0.1% formic acid (LC-MS grade, Sigma-Aldrich, Saint Louis, MO, USA); and-mobile phase (B)—methanol (LC-MS grade, Sigma-Aldrich, Saint Louis, MO, USA) with a constant flow rate of 300 μL/min. The gradient profile was as follows: 0–2 min, 5% B; 2–10 min, linear rise to 70% B; 10–12 min, linear increase to 100% B; 12–17 min, hold at 100% B; 17.0–17.1 min, linear reduction to 1% B; and 17.1–19 min, hold at 1% B. The mass spectrometer was equipped with a heated electrospray ionization (HESI) source and operated in negative ion mode, tuned with 1 ppm standard solutions of each analyte in methanol. Key mass spectrometry settings were as follows: HESI operating in negative ion mode, spray voltage at 2500 V, auxiliary gas pressure of 10 a.u., sheath gas at 50 psi, sweep gas at 1 a.u., ion transfer tube at 325 °C, vaporizer temperature at 350 °C, and dwell time at 100 ms. Q1 resolution set to 1 Da and Q3 resolution to 0.4 Da, with CID gas (Ar) at 2.0 mTorr. Selected reaction monitoring (SRM) targeted deprotonated ions for each polyphenol ([M-H]-), with specific SRM transitions detailed in Table 2. Quantification was achieved by integrating the peak areas of monitored transitions and the following pure standards for each compound: apigenin 7-glucoside, apigenin, quercetin, gallic acid, l-mandelic acid, chlorogenic acid, hydroxycinnamic acid, kaempferol, caffeic acid, vanillic acid, catechin, rutin, coumaric acid, syringic acid, gentisic acid, ferulic acid, luteonin, and resveratrol.

Phenolic compound quantification was performed using an external calibration approach. A methanolic solution containing 5 ppm of each standard was prepared, followed by a series of additional calibration solutions of 1 ppm, 500 ppb, 250 ppb, 100 ppb, and 50 ppb for each analyte. The linearity correlation coefficient (R^2^) was 0.99. Data were analyzed using the Quan/Qual Browser and Trace Finder (Thermo Fisher Scientific), and each point on the calibration graph corresponded to the average of three independent injections.

### 4.5. Reducing Capacity and Radical Scavenging Test

#### 4.5.1. Sample Preparation

For the FRAP, DPPH, and ABTS assays, chitosan was solubilized in 2 M acetic acid to obtain a 2% (*w*/*v*) solution; the ethanolic extract of astaxanthin was used as is, and the dry residue of phenolic extract was resuspended in 1 mL of 5 mM phosphate-buffered saline (PBS) at pH of 7.4.

#### 4.5.2. Reducing Capacity Test

The ferric ion reducing antioxidant power (FRAP) assay was performed following Saxena et al.’s method [109], with minor modifications. Samples were diluted in 1 mL of 0.2 M phosphate buffer (pH 6.6) and mixed with 1 mL of 1% potassium ferricyanide. The resulting blend was then incubated at 50 °C for 20 min before adding 1 mL of 10% trichloroacetic acid and combining thoroughly with a vortex mixer. This solution was then centrifuged for 10 min at 1000× *g*, and a 0.5 mL portion of the supernatant was combined with 0.5 mL of distilled water and 0.1 mL of 0.1% FeCl_3_ solution. Absorbance was measured at 700 nm. Ascorbic acid (0–100 μg/mL) was used as a reference compound, and results are expressed in milligrams of ascorbic acid equivalents (AAE) per 1 g of sample.

#### 4.5.3. Radical Scavenging Activity Assay

2,2′-Azino-bis (3-ethylbenzothiazoline-6-sulfonic acid) (ABTS^•+^) radical cation was prepared following Re et al.’s method [110]. Stock solutions were prepared with 7.0 mM ABTS diazonium salt and 140 mM potassium persulfate in water. The working solution was made by combining 1 mL of ABTS solution and 18 μL of potassium persulfate and allowing reaction to occur at 25 °C for 18 h in the dark. A 900 μL aliquot of the resulting solution was then diluted with 100 μL of phosphate saline buffer (PBS) to achieve an absorbance of 0.700 at 734 nm. Portions of appropriately diluted sample (100 μL) were mixed with 900 μL of the ABTS^•+^ solution and allowed to react for 15 min in the dark. The absorbance was then measured at 734 nm.

The free radical scavenging activity of 2,2-Diphenyl-1-picrylhydrazyl (DPPH^•^) was assessed using the method described by Brand-Williams et al. [111]. Aliquots (100 μL) of appropriately diluted samples were mixed with 900 μL of DPPH^•^ ethanol solution (1 × 10^−4^ mol/L), and absorbance was measured at 515 nm following 30 min incubation at room temperature in the dark. The ABTS^•+^ and DPPH^•^ radical scavenging activities of the samples were compared to Trolox, a water-soluble analog of vitamin E, with results expressed as μmol of Trolox equivalents (TE) per g of sample.

### 4.6. Cell Toxicity Assay

The cytotoxicity of astaxanthin and polyphenol extracts on tumoral and differentiated cells was determined using 3-(4,5-dimethylthiazol-2-yl)-2,5 diphenyl tetrazolium bromide (MTT) assays for cellular viability analysis, as already reported [112]. HepG2 liver cancer cells and CaCo-2 colon cancer cells taken from laboratory stocks were grown in glutamine-containing Dulbecco’s Modified Eagle Medium (DMEM) (GibcoTM, Fisher Scientific, Segrate, Italy) supplemented with 10% heat-inactivated fetal bovine serum (Sigma) and antibiotics (100 U/mL penicillin and 100 g/mL streptomycin; Capricorn Scientific GmbH, Ebsdorfergrund, Germany) at 37 °C under 5% CO_2_ in humidified air. In addition to cancerous CaCo-2 cells, cultures of the same cells spontaneously differentiated in enterocytes were prepared according to Cicio et al. [113] and tested in parallel. Cells in exponential growth were seeded at a concentration of 8000/well (HepG2) and 12000/well (CaCo-2) in 96-well plates, left to adhere overnight and cultured in control conditions or exposed to varying concentrations of either astaxanthin or polyphenol extract for 24 h. After the addition of 3-(4,5-dimethylthiazol-2-yl)-2,5 diphenyl tetrazolium bromide (MTT; Merck, Milano, Italy) and cell solubilization, the optical density of the dissolved formazan was probed at 550 nm. Cell viabilities were expressed as a relative percentage compared to the controls.

The half maximal inhibitory concentrations (IC_50_) for astaxanthin and polyphenol extract were quantitated using the ED50 PLUS V1.0 software available online at https://www.sciencegateway.org/protocols/cellbio/drug/data/ed50v10.xls (accessed on 10 October 2024). The astaxanthin extract was prepared by evaporating the extraction solvent under reduced pressure using a rotary evaporator. The dry residue was resuspended in a known volume of absolute ethanol to obtain an astaxanthin solution of 2 mg/mL. The polyphenol extract was prepared as described previously (see Section 4.5.1). Both extracts were filtered under a laminar flow hood using a 0.22 µm membrane filter (Millex^®^, Merck Millipore, Darmstadt, Germany).

### 4.7. Statistical Analysis

The experiments were conducted in triplicate (*n* ≥ 3), and the results are presented as mean values ± standard deviation (SD) or standard error of the mean (s.e.m). One-way analysis of variance (ANOVA) followed by Tukey’s test, with a significance level set at *p* < 0.05, was used to determine significant differences.

## 5. Conclusions

Bioactive molecules, including chitosan, astaxanthin, and polyphenols, with multiple applications in various sectors, were extracted from the exoskeleton of *C. sapidus* captured in Sicily. These compounds have demonstrated significant radical scavenging activity, reducing capacity, and anticancer properties, highlighting their potential not only in the nutraceutical field but also in pharmaceuticals and biotechnology. In particular, chitosan and astaxanthin, known for their ability to combat oxidative stress, could be used in various fields ranging from cosmetics to regenerative medicine. Likewise, polyphenolic compounds extracted from the exoskeleton of *C. sapidus* exhibit antioxidant activity and could find applications in dietary supplements or as natural additives in a range of products. The antitumor effect shown in vitro by astaxanthin and phenolic preparation warrant further investigation into their potential role in cancer treatment. These findings not only emphasize the importance of the species in the context of human consumption but also suggest that the use of this invasive species for the extraction of bioactive molecules could provide an innovative solution to control its spread. In addition to creating new sectors of employment and economic value, this practice would reduce the environmental impact caused by the uncontrolled proliferation of *C. sapidus* in Mediterranean waters.

## Figures and Tables

**Table 1:** Bio-phenolic compounds detected in *C. sapidus* exoskeleton.

Bio-Phenol	Calibration Equation	Concentration (mg/100 mL)
Coumaric acid	Y = −10,089 + 6127X	0.20
Ferulic acid	Y = 1614 + 1181X	0.12
Gentisic acid	Y = −58,051.4 + 3441.27X	<LOD
Luteolin	Y = −1577 + 10,979X	0.02
Mandelic acid	Y = −25,181.1 + 488.34X	1.08
Rutin	Y = −61,324.9 + 2962.87X	0.09
Trans-OH-Cynnamic acid	Y = −121.915 + 6685.59X	0.08

**Table 2:** UPLC-MS parameters and limit of quantification for phenolic compounds identified in hydromethanolic extracts of *C. sapidus*.

	Precursor Ion (*m*/*z*) [M-H]^−^	Product Ion (*m*/*z*)	Collision Energy (V)	RF Lens (V)
Gallic Acid	169	79	24	101
	169	125	14	101
Vanillic Acid	177	123	20	105
	177	152	20	105
Ferulic Acid	193	134	15	99
	193	178	13	99
Chlorogenic Acid	353	179	45	180
	353	191	45	180
Catechin	289	203	20	147
	289	245	15	147
Mandelic Acid	151	77	18	65
	151	107	10	65
Gentisic Acid	153	108	22	90
	153	109	14	90
Syringic Acid	197	153	12	100
	197	182	14	100
Caffeic Acid	179	107	25	101
	179	135	16	103
Trans-OH-Cynnamic	163	93	31	90
	163	119	14	90
Rutin	609	271	60	299
	609	300	38	299
Resveratrol	227	143	27	156
	227	185	20	156
Apigenin-7Glu	433	269	20	123
	433	271	20	123
Quercetin	301	151	18	166
	301	179	21	166
Kaempferol	285	202	20	195
	285	239	29	195
Hydroxytyrosol	153	95	21	97
	153	123	14	97
Cumaric Acid	163	93	31	91
	163	119	13	91
Luteolin	285	133	35	187
	285	175	26	187
Apigenin	269	117	35	178
	269	151	25	178

## Data Availability

Data will provide on request.

## References

[1] Guerin J.L., Stickle W.B. (1992). Effects of salinity gradients on the tolerance and bioenergetics of juvenile blue crabs (*Callinectes sapidus*) from waters of different environmental salinities. Mar. Biol..

[2] Nehring S., Galil B.S., Clark P.F., Carlton J.T. (2011). Invasion history and success of the American blue crab *Callinectes sapidus* in European and adjacent waters. In the Wrong Place-Alien Marine Crustaceans: Distribution, Biology and Impacts.

[3] Laughlin R.A. (1982). Feeding habits of the blue crab, *Callinectes sapidus* Rathbun, in the Apalachicola estuary, Florida. Bull. Mar. Sci..

[4] Ortega-Jiménez E., Cuesta J.A., Laiz I., González-Ortegón E. (2024). Diet of the Invasive Atlantic Blue Crab *Callinectes sapidus* Rathbun, 1896 (Decapoda, Portunidae) in the Guadalquivir Estuary (Spain). Estuaries Coasts.

[5] Mancinelli G., Bardelli R., Zenetos A. (2021). A global occurrence database of the Atlantic blue crab *Callinectes sapidus*. Sci. Data.

[6] Zenetos A., Galanidi M. (2020). Mediterranean non indigenous species at the start of the 2020s: Recent changes. Mar. Biodivers. Rec..

[7] Giordani-Soika A. (1951). Il *Neptunus pelagicus* (L.) nell’alto Adriatico. Natura.

[8] Mizzan L. (1993). Presence of swimming crabs of the genus Callinectes (Stimpson) (Decapoda, Portunidae) in the Venice Lagoon (North Adriatic Sea-Italy). First record of Callinectes danae Smith in European waters. Boll. Mus. Civ. Stor. Nat. Venezia.

[9] Mancinelli G., Chainho P., Cilenti L., Falco S., Kapiris K., Katselis G., Ribeiro F. (2017). On the Atlantic blue crab (*Callinectes sapidus* Rathbun 1896) in southern European coastal waters: Time to turn a threat into a resource?. Fish. Res..

[10] Longmire K.S., Seitz R.D., Seebo M.S., Brill R.W., Lipcius R.N. (2022). Biological responses of the predatory blue crab and its hard clam prey to ocean acidification and low salinity. Mar. Ecol. Prog. Ser..

[11] Galil B.S. (2000). A Sea Under Siege—Alien Species in the Mediterranean. Biol. Invasions.

[12] Azzurro E., Bonanomi S., Chiappi M., De Marco R., Luna G.M., Cella M., Guicciardi S., Tiralongo F., Bonifazi A., Strafella P. (2024). Uncovering unmet demand and key insights for the invasive blue crab (*Callinectes sapidus*) market before and after the Italian outbreak: Implications for policymakers and industry stakeholders. Mar. Policy.

[13] Cavaliere A., Berdar A. (1975). Presenza di *Callinectes sapidus* Rathbun (Decapoda, Brachyura) nello Stretto di Messina. Boll. Pesca Piscic. Idrobiol..

[14] Castriota L., Falautano M. (2021). Reviewing the invasion history of the blue crab *Callinectes sapidus* (Portunidae) in Sicily (Central Mediterranean): An underestimated alien species. Ann. Ser. Hist. Nat..

[15] Bardelli R., Mancinelli G., Mazzola A., Vizzini S. (2023). The Atlantic blue crab *Callinectes sapidus* spreading in the Tyrrhenian sea: Evidence of an established population in the Stagnone di Marsala (Sicily, southern Italy). Nase More.

[16] Di Martino V., Stancanelli B. (2021). Mass mortality event of *Callinectes sapidus* Rathbun 1896 in a coastal pond of the protect area of Vendicari in summer 2020 (SE Sicily). J. Sea Res..

[17] Vecchioni L., Russotto S., Arculeo M., Marrone F. (2022). On the occurrence of the invasive Atlantic blue crab *Callinectes sapidus* Rathbun 1896 (Decapoda: Brachyura: Portunidae) in Sicilian inland waters. Nat. Hist. Sci..

[18] Vecchioni L., Faraone F.P., Stoch F., Arculeo M., Marrone F. (2022). Diversity and Distribution of the Inland Water Decapods of Sicily (Crustacea, Malacostraca). Diversity.

[19] Vella A., Giarrusso E., Monaco C., Mifsud C.M., Darmanin S.A., Raffa A., Tumino C., Peri I., Vella N. (2023). New Records of *Callinectes sapidus* (Crustacea, Portunidae) from Malta and the San Leonardo River Estuary in Sicily (Central Mediterranean). Diversity.

[20] Khamassi F., Bahri W.R., Bhouri A.M., Chaffai A., Ghanem R., Souissi J.B. (2022). Biochemical composition, nutritional value and socio-economic impacts of the invasive crab *Callinectes sapidus* Rathbun, 1896 in the central Mediterranean Sea. Mediterr. Mar. Sci..

[21] Küçükgülmez A., Celik M., Yanar Y., Ersoy B., Çikrikçi M. (2006). Proximate composition and mineral contents of the blue crab (*Callinectes sapidus*) breast meat, claw meat and hepatopancreas. Int. J. Food Sci. Technol..

[22] Tufan B. (2023). Biochemical composition of different sex and body parts of blue crabs (*Callinectes sapidus*) caught from the middle Black Sea coast. Mar. Sci. Technol. Bull..

[23] Öndes F., Gökçe G. (2021). Distribution and fishing of the invasive blue crab (*Callinectes sapidus*) in Turkey based on local ecological knowledge of fishermen. J. Anatol. Environ. Anim. Sci..

[24] Official Journal of the Italian Republic, General Series. 2024, 165. https://www.gazzettaufficiale.it/eli/gu/2024/01/26/21/sg/pdf.

[25] Baron R.D., Pérez L.L., Salcedo J.M., Córdoba L.P., Do Amaral Sobral P.J. (2017). Production and characterization of films based on blends of chitosan from blue crab (*Callinectes sapidus*) waste and pectin from Orange (*Citrus sinensis Osbeck*) peel. Int. J. Biol. Macromol..

[26] Chung H.Y., Cadwallader K.R. (1993). Volatile components in blue crab (*Callinectes sapidus*) meat and processing by-product. J. Food Sci..

[27] Arena R., Renda G., Ottaviani A.G., Debeaufort F., Messina C.M., Santulli A. (2024). Valorization of the Invasive Blue Crabs (*Callinectes sapidus*) in the Mediterranean: Nutritional Value, Bioactive Compounds and Sustainable By-Products Utilization. Mar. Drugs.

[28] Tamburini E. (2024). The blue treasure: Comprehensive biorefinery of blue crab (*Callinectes sapidus*). Foods.

[29] Mathew G.M., Mathew D.C., Sukumaran R.K., Sindhu R., Huang C.C., Binod P., Sirohi R., Kim S.H., Pandey A. (2020). Sustainable and eco-friendly strategies for shrimp shell valorization. Environ. Pollut..

[30] Zhang Z., Ma Z., Song L., Farag M.A. (2023). Maximizing crustaceans (shrimp, crab, and lobster) by-products value for optimum valorization practices: A comparative review of their active ingredients, extraction, bioprocesses and applications. J. Adv. Res..

[31] Hülsey M.J. (2018). Shell biorefinery: A comprehensive introduction. Green Energy Environ..

[32] Zhang S.Y., Zhao Y.Q., Wang Y.M., Yang X.R., Chi C.F., Wang B. (2022). Gelatins and antioxidant peptides from Skipjack tuna (*Katsuwonus pelamis*) skins: Purification, characterization, and cytoprotection on ultraviolet-A injured human skin fibroblasts. Food Biosci..

[33] Zhang Z., Wang Y.M., Qiu Y.T., Chi C.F., Luo H.Y., Wang B. (2022). Gelatin from cartilage of siberian sturgeon (*Acipenser baerii*): Preparation, characterization, and protective function on ultraviolet-A-Injured human skin fibroblasts. Front. Mar. Sci..

[34] Hu Y.-D., Xi Q.-H., Kong J., Zhao Y.-Q., Chi C.-F., Wang B. (2023). Angiotensin-I-Converting Enzyme (ACE)-Inhibitory Peptides from the Collagens of Monkfish (*Lophius litulon*) Swim Bladders: Isolation, Characterization, Molecular Docking Analysis and Activity Evaluation. Mar. Drugs.

[35] Pan X., Zhao Y.Q., Hu F.Y., Wang B. (2016). Preparation and identification of antioxidant peptides from protein hydrolysate of skate (*Raja porosa*) cartilage. J. Funct. Foods.

[36] Chi C.F., Wang B., Wang Y.M., Zhang B., Deng S.G. (2015). Isolation and characterization of three antioxidant peptides from protein hydrolysate of bluefin leatherjacket (*Navodon septentrionalis*) heads. J. Funct. Foods.

[37] Elieh-Ali-Komi D., Hamblin M.R. (2016). Chitin and chitosan: Production and application of versatile biomedical nanomaterials. Int. J. Adv. Res..

[38] Younes I., Rinaudo M. (2015). Chitin and chitosan preparation from marine sources. Structure, properties and applications. Mar. Drugs.

[39] Wang W., Xue C., Mao X. (2020). Chitosan: Structural modification, biological activity and application. Int. J. Biol. Macromol..

[40] Pita-López M.L., Fletes-Vargas G., Espinosa-Andrews H., Rodriguez-Rodriguez R. (2021). Physically cross-linked chitosan-based hydrogels for tissue engineering applications: A state-of-the-art review. Eur. Polym. J..

[41] Al-Manhel A.J., Al-Hilphy A.R.S., Niamah A.K. (2018). Extraction of chitosan, characterisation and its use for water purification. J. Saudi Soc. Agric. Sci..

[42] Kashyap P.L., Xiang X., Heiden P. (2015). Chitosan nanoparticle-based delivery systems for sustainable agriculture. Int. J. Biol. Macromol..

[43] El-Aidie S. (2018). A Review on Chitosan: Ecofriendly Multiple Potential Applications in the Food Industry. Int. J. Life Sci. Res..

[44] Edo G.I., Yousif E., Al-Mashhadani M.H. (2024). Chitosan: An overview of biological activities, derivatives, properties, and current advancements in biomedical applications. Carbohydr. Res..

[45] Azuma K., Osaki T., Minami S., Okamoto Y. (2015). Anticancer and anti-inflammatory properties of chitin and chitosan oligosaccharides. J. Funct. Biomater..

[46] Ambati R.R., Phang S.M., Ravi S., Aswathanarayana R.G. (2014). Astaxanthin: Sources, extraction, stability, biological activities and its commercial applications—A review. Mar. Drugs.

[47] Maoka T. (2020). Carotenoids as natural functional pigments. J. Nat. Med..

[48] Sztretye M., Dienes B., Gönczi M., Czirják T., Csernoch L., Dux L., Szentesi P., Keller-Pintér A. (2019). Astaxanthin: A potential mitochondrial-targeted antioxidant treatment in diseases and with aging. Oxidative Med. Cell. Longev..

[49] Miki W. (1991). Biological functions and activities of animal carotenoids. Pure Appl. Chem..

[50] Zhang L., Wang H. (2015). Multiple mechanisms of anti-cancer effects exerted by astaxanthin. Mar. Drugs.

[51] Stachowiak B., Szulc P. (2021). Astaxanthin for the food industry. Molecules.

[52] Pashkow F.J., Watumull D.G., Campbell C.L. (2008). Astaxanthin: A novel potential treatment for oxidative stress and inflammation in cardiovascular disease. Am. J. Cardiol..

[53] Official Journal of the European Union. http://data.europa.eu/eli/reg_impl/2015/1415/oj.

[54] Di Lorenzo C., Colombo F., Biella S., Stockley C., Restani P. (2021). Polyphenols and human health: The role of bioavailability. Nutrients.

[55] Lacikova L., Jancova M., Muselik J., Masterova I., Grancai D., Fickova M. (2009). Antiproliferative, Cytotoxic, Antioxidant Activity and Polyphenols Contents in Leaves of Four *Staphylea* L. Species. Molecules.

[56] Shahidi F., Hossain A. (2022). Preservation of aquatic food using edible films and coatings containing essential oils: A review. Crit. Rev. Food Sci. Nutr..

[57] Onodenalore A.C., Hossain A., Banoub J., Shahidi F. (2024). Unique Heterocyclic Phenolic Compounds from Shrimp (*Pandalus borealis*) and Beyond. Food Prod. Process. Nutr..

[58] Pereira U.C., Chagas Barros R.G., Santana Andrade J.K., De Oliveira C.S., Gualberto N.C., Narain N. (2020). Effect of In Vitro Gastrointestinal Digestion on Bioaccessibility of Phenolic Compounds and Antioxidant Capacity of Crustaceans Residues with Potential Antidiabetic Impact. LWT.

[59] Abd El-Ghany M.N., Hamdi S.A., Elbaz R.M., Aloufi A.S., El Sayed R.R., Ghonaim G.M., Farahat M.G. (2023). Development of a microbial-assisted process for enhanced astaxanthin recovery from crab exoskeleton waste. Fermentation.

[60] Vidal J.L., Jin T., Lam E., Kerton F., Moores A. (2022). Blue is the new green: Valorization of crustacean waste. Curr. Res. Green Sustain..

[61] Kiehbadroudinezhad M., Hosseinzadeh-Bandbafha H., Varjani S., Wang Y., Peng W., Pan J., Aghbashlo M., Tabatabaei M. (2023). Marine shell-based biorefinery: A sustainable solution for aquaculture waste valorization. Renew. Energy.

[62] Rossi N., Grosso C., Delerue-Matos C. (2024). Shrimp Waste Upcycling: Unveiling the Potential of Polysaccharides, Proteins, Carotenoids, and Fatty Acids with Emphasis on Extraction Techniques and Bioactive Properties. Mar. Drugs.

[63] Muzzarelli R.A., Sarmento B., das Neves J. (2012). Chemical and technological advances in chitins and chitosans useful for the formulation of biopharmaceuticals. Chitosan-Based Systems for Biopharmaceuticals: Delivery, Targeting and Polymer Therapeutics.

[64] Isa M.T., Abdulkarim A.Y., Bello A., Bello T.K., Adamu Y. (2024). Synthesis and characterization of chitosan for medical applications: A review. J. Biomater. Appl..

[65] Metin C., Alparslan Y., Baygar T., Baygar T. (2019). Physicochemical, Microstructural and Thermal Characterization of Chitosan from Blue Crab Shell Waste and Its Bioactivity Characteristics. J. Polym. Environ..

[66] Kaya M., Dudakli F., Asan-Ozusaglam M., Cakmak Y.S., Baran T., Mentes A., Erdogan S. (2016). Porous and nanofiber α-chitosan obtained from blue crab (*Callinectes sapidus*) tested for antimicrobial and antioxidant activities. LWT-Food Sci. Technol..

[67] Wenling C., Duohui J., Jiamou L., Yandao G., Nanming Z., Xiufang Z. (2005). Effects of the Degree of Deacetylation on the Physicochemical Properties and Schwann Cell Affinity of Chitosan Films. J. Biomater. Appl..

[68] Muzzarelli R.A.A. (2011). Chitosan composites with inorganics, morphogenetic proteins and stem cells, for bone regeneration. Carbohydr. Polym..

[69] Pal J., Hari O.V., Vijay K.M., Satyendra K.M., Deepayan R., Jitendra K. (2014). Biological method of chitin extraction from shrimp waste an eco-friendly low-cost technology and its advanced application. Int. J. Fish. Aquat. Stud..

[70] Šimat V., Rathod N.B., Čagalj M., Hamed I., Generalić Mekinić I. (2022). Astaxanthin from Crustaceans and Their Byproducts: A Bioactive Metabolite Candidate for Therapeutic Application. Mar. Drugs.

[71] Felix-Valenzuela L.E., Higuera-ciapara I.N., Goycoolea-valencia F.R., Argüelles-Monal W.A. (2001). Supercritical CO_2_/ethanol extraction of astaxanthin from blue crab (*Callinectes sapidus*) shell waste. J. Food Process Eng..

[72] Antunes-Valcareggi S.A., Ferreira S.R., Hense H. (2017). Enzymatic hydrolysis of blue crab (*Callinectes sapidus*) waste processing to obtain chitin, protein, and astaxanthin-enriched extract. Int. J. Environ. Agric. Res..

[73] Montoya J.M., Mata S., Acosta J., Cabrera B., López-Valdez L., Reyes C., Barrales-Cureño H. (2021). Obtaining of astaxanthin from crab exosqueletons and shrimp head shells. Biointerface Res. Appl. Chem..

[74] Babin A., Motreuil S., Teixeira M., Bauer A., Rigaud T., Moreau J., Moret Y. (2020). Origin of the Natural Variation in the Storage of Dietary Carotenoids in Freshwater Amphipod Crustaceans. PLoS ONE.

[75] Yanar Y., Çelik M., Yanar M. (2004). Seasonal Changes in Total Carotenoid Contents of Wild Marine Shrimps (*Penaeus semisulcatus* and *Metapenaeus monoceros*) Inhabiting the Eastern Mediterranean. Food Chem..

[76] Yusoff I.M., Taher Z.M., Rahmat Z., Chua L.S. (2022). A review of ultrasound-assisted extraction for plant bioactive compounds: Phenolics, flavonoids, thymols, saponins and proteins. Int. Food Res..

[77] Ojha K.S., Aznar R., O’Donnell C., Tiwari B.K. (2020). Ultrasound technology for the extraction of biologically active molecules from plant, animal and marine sources. TrAC Trends Anal. Chem..

[78] Panagiotakopoulos I., Karantonis H.C., Kartelias I.G., Nasopoulou C. (2023). Ultrasonic-Assisted Extraction of Astaxanthin from Shrimp By-Products Using Vegetable Oils. Mar. Drugs.

[79] Denga M., Qu Y., Wu T., Na Y., Liang N., Zhao L. (2024). Amino acid-based natural deep eutectic solvent combined with ultrasonic extraction: Green extraction of astaxanthin from shrimp shells. Biomass Convers. Biorefin..

[80] Sayem A.S.M., Ahmed T., Mithun M.U.K., Rashid M., Rana M.R. (2024). Optimising ultrasound-assisted extraction conditions for maximising phenolic, flavonoid content and antioxidant activity in hog plum peel and seed: A response surface methodology approach. J. Agric. Food Res..

[81] Maia M.L., Grosso C., Barroso M.F., Silva A., Delerue-Matos C., Domingues V.F. (2023). Bioactive Compounds of Shrimp Shell Waste from *Palaemon serratus* and *Palaemon varians* from Portuguese Coast. Antioxidants.

[82] Longo F., Di Gaudio F., Attanzio A., Marretta L., Luparello C., Indelicato S., Bongiorno D., Barone G., Tesoriere L., Giardina I.C. (2024). Bioactive Molecules from the Exoskeleton of *Procambarus clarkii*: Reducing Capacity, Radical Scavenger, and Antitumor and Anti-Inflammatory Activities. Biomolecules.

[83] Măgerușan Ș.E., Hancu G., Rusu A. (2023). A Comprehensive Bibliographic Review Concerning the Efficacy of Organic Acids for Chemical Peels Treating Acne Vulgaris. Molecules.

[84] Sun T., Qin Y., Xie J., Xue B., Zhu Y., Wu J., Bian X., Li X. (2018). Antioxidant Activity of Oligochitosan Maillard Reaction Products Using Oligochitosan as the Amino or Carbonyl Groups Donors. Int. J. Food Prop..

[85] Pu S., Li J., Sun L., Zhong L., Ma Q. (2019). An in vitro comparison of the antioxidant activities of chitosan and green synthesized gold nanoparticles. Carbohydr. Polym..

[86] Müller L., Fröhlich K., Böhm V. (2011). Comparative antioxidant activities of carotenoids measured by ferric reducing antioxidant power (FRAP), ABTS bleaching assay (αTEAC), DPPH assay and peroxyl radical scavenging assay. Food Chem..

[87] Kumar S., Kumar R., Diksha, Kumari A., Panwar A. (2022). Astaxanthin: A super antioxidant from microalgae and its therapeutic potential. J. Basic Microbiol..

[88] Liu X., Luo Q., Rakariyatham K., Cao Y., Goulette T., Liu X., Xiao H. (2016). Antioxidation and anti-ageing activities of different stereoisomeric astaxanthin in vitro and in vivo. J. Funct. Foods.

[89] Guerin M., Huntley M.E., Olaizola M. (2003). *Haematococcus* Astaxanthin: Applications for Human Health and Nutrition. Trends Biotechnol..

[90] Olszowy M. (2019). What Is Responsible for Antioxidant Properties of Polyphenolic Compounds from Plants?. Plant Physiol. Biochem..

[91] Messina C.M., Manuguerra S., Renda G., Santulli A. (2019). Biotechnological Applications for the Sustainable Use of Marine By-products: In Vitro Antioxidant and Pro-apoptotic Effects of Astaxanthin Extracted with Supercritical CO_2_ from *Parapeneus longirostris*. Mar. Biotechnol..

[92] Tan Y., Ye Z., Wang M., Manzoor M.F., Aadil R.M., Tan X., Liu Z. (2021). Comparison of Different Methods for Extracting the Astaxanthin from *Haematococcus pluvialis*: Chemical Composition and Biological Activity. Molecules.

[93] Wayakanon K., Rueangyotchanthana K., Wayakanon P., Suwannachart C. (2018). The inhibition of Caco-2 proliferation by astaxanthin from *Xanthophyllomyces dendrorhous*. J. Med. Microbiol..

[94] Sadi G., Kaya A., Yalcin H.A., Emsen B., Kocabas A., Kartal D.I., Altay A. (2016). Wild Edible Mushrooms from Turkey as Possible Anticancer Agents on HepG2 Cells Together with Their Antioxidant and Antimicrobial Properties. Int. J. Med. Mushrooms.

[95] Janicke B., Hegardt C., Krogh M., Önning G., Åkesson B., Cirenajwis H.M., Oredsson S.M. (2011). The Antiproliferative Effect of Dietary Fiber Phenolic Compounds Ferulic Acid and *p* -Coumaric Acid on the Cell Cycle of Caco-2 Cells. Nutr. Cancer.

[96] Karakurt S., Abuşoğlu G., Arituluk Z.C. (2020). Comparison of Anticarcinogenic Properties of Viburnum Opulus and Its Active Compound P-Coumaric Acid on Human Colorectal Carcinoma. Turk. J. Biol..

[97] Wang J., Lai X., Yuan D., Liu Y., Wang J., Liang Y. (2022). Effects of Ferulic Acid, a Major Component of Rice Bran, on Proliferation, Apoptosis, and Autophagy of HepG2 Cells. Food Res. Int..

[98] Janda E., Martino C., Riillo C., Parafati M., Lascala A., Mollace V., Boutin J.A. (2021). Apigenin and Luteolin Regulate Autophagy by Targeting NRH-Quinone Oxidoreductase 2 in Liver Cells. Antioxidants.

[99] Ben Sghaier M., Pagano A., Mousslim M., Ammari Y., Kovacic H., Luis J. (2016). Rutin Inhibits Proliferation, Attenuates Superoxide Production and Decreases Adhesion and Migration of Human Cancerous Cells. Biomed. Pharmacother..

[100] Yu M.-H., Tsai M.-C., Wang C.-C., Wu S.-W., Chang Y.-J., Wu C.-H., Wang C.-J. (2021). Mulberry Leaf Polyphenol Extract and Rutin Induces Autophagy Regulated by P53 in Human Hepatoma HepG2 Cells. Pharmaceuticals.

[101] Yuan W., Zheng B., Li T., Liu R.H. (2022). Quantification of Phytochemicals, Cellular Antioxidant Activities and Antiproliferative Activities of Raw and Roasted American Pistachios (*Pistacia vera* L.). Nutrients.

[102] Mauro M., Pinto P., Settanni L., Puccio V., Vazzana M., Hornsby B.L., Fabbrizio A., Di Stefano V., Barone G., Arizza V. (2022). Chitosan Film Functionalized with Grape Seed Oil—Preliminary Evaluation of Antimicrobial Activity. Sustainability.

[103] Brugnerotto J., Lizardi J., Goycoolea F.M., Argüelles-Monal W., Desbrières J., Rinaudo M. (2001). An Infrared Investigation in Relation with Chitin and Chitosan Characterization. Polymer.

[104] Hu J., Lu W., Lv M., Wang Y., Ding R., Wang L. (2019). Extraction and Purification of Astaxanthin from Shrimp Shells and the Effects of Different Treatments on Its Content. Rev. Bras. Farmacogn..

[105] Sharayei P., Azarpazhooh E., Zomorodi S., Einafshar S., Ramaswamy H.S. (2021). Optimization of ultrasonic-assisted extraction of astaxanthin from green tiger (*Penaeus semisulcatus*) shrimp shell. Ultrason. Sonochem..

[106] Singleton V.L., Orthofer R., Lamuela-Raventos R.M., Packer L. (1999). Analysis of Total Phenols and Other Oxidation Substrates and Antioxidants by Means of Folin-Ciocalteu Reagent. Methods in Enzymology.

[107] Attanzio A., D’Anneo A., Pappalardo F., Bonina F.P., Livrea M.A., Allegra M., Tesoriere L. (2019). Phenolic Composition of Hydrophilic Extract of Manna from Sicilian *Fraxinus angustifolia* Vahl and Its Reducing, Antioxidant and Anti-Inflammatory Activity In Vitro. Antioxidants.

[108] Indelicato S., Houmanat K., Bongiorno D., Ejjilani A., Hssaini L., Razouk R., Charafi J., Ennahli S., Hanine H. (2023). Freeze Dried Pomegranate Juices of Moroccan Fruits: Main Representative Phenolic Compounds. J. Sci. Food Agric..

[109] Saxena S., Gautam S., Sharma A. (2010). Physical, Biochemical and Antioxidant Properties of Some Indian Honeys. Food Chem..

[110] Re R., Pellegrini N., Proteggente A., Pannala A., Yang M., Rice-Evans C. (1999). Antioxidant activity applying an improved ABTS radical cation decolorization assay. Free Radic. Biol. Med..

[111] Brand-Williams W., Cuvelier M.E., Berset C. (1995). Use of a Free Radical Method to Evaluate Antioxidant Activity. LWT—Food Sci. Technol..

[112] Luparello C., Branni R., Abruscato G., Lazzara V., Drahos L., Arizza V., Mauro M., Di Stefano V., Vazzana M. (2022). Cytotoxic Capability and the Associated Proteomic Profile of Cell-Free Coelomic Fluid Extracts from the Edible Sea Cucumber *Holothuria tubulosa* on HepG2 Liver Cancer Cells. EXCLI J..

[113] Cicio A., Sut S., Dall’Acqua S., Bruno M., Luparello C., Serio R., Zizzo M.G. (2023). Chemical Characterization and Cytotoxic and Antioxidant Activity Evaluation of the Ethanol Extract from the Bulbs of *Pancratium maritimun* Collected in Sicily. Molecules.

